# Prevalence of *Clostridioides difficile* Infection in Critically Ill Patients with Extreme Leukocytosis and Diarrhea

**DOI:** 10.3390/idr13010003

**Published:** 2021-01-01

**Authors:** Bijan Teja, Nafeesa Alibhai, Gordon D. Rubenfeld, Linda R. Taggart, Naheed Jivraj, Sameer A. Hirji, Brian P. O’Gara, Shahzad Shaefi

**Affiliations:** 1 Interdepartmental Division of Critical Care Medicine, University of Toronto, Toronto, ON M5B 1T8, Canada; gordon.rubenfeld@sunnybrook.ca (G.D.R.); naheed@jivraj.ca (N.J.); 2 Institute of Health Policy, Management and Evaluation, University of Toronto, Toronto, ON M5T 3M6, Canada; 3 Honors Integrated Sciences, University of British Columbia, Vancouver, BC V6T 1Z2, Canada; alibhai.nafeesa@gmail.com; 4 Division of Infectious Diseases, Department of Medicine, St. Michael’s Hospital, Toronto, ON M5B 1W8, Canada; linda.taggart@unityhealth.to; 5 Department of General Surgery, Brigham and Women’s Hospital, Harvard Medical School, Boston, MA 02115, USA; shirji@partners.org; 6 Department of Anesthesia, Critical Care and Pain Medicine, Beth Israel Deaconess Medical Center, Harvard Medical School, Boston, MA 02215, USA; bpogara@bidmc.harvard.edu (B.P.O.); sshaefi@bidmc.harvard.edu (S.S.)

**Keywords:** *Clostridium difficile*, *Clostridioides difficile*, leukocytosis, sepsis, critical care medicine

## Abstract

While early empiric antibiotic therapy is beneficial for patients presenting with sepsis, the presentation of sepsis from *Clostridioides difficile* (formerly *Clostridium difficile*) infection (CDI) has not been well studied in large cohorts. We sought to determine whether the combination of extreme leukocytosis and diarrhea was strongly predictive of CDI in a cohort of 8659 patients admitted to the intensive care unit. We found that CDI was present in 15.0% (95% CI, 12.1–18.3%) of patients with extreme leukocytosis and diarrhea and that mortality for those with CDI, diarrhea, and extreme leukocytosis was 33.8% (95% CI, 23.2–44.3%). These data support consideration of empiric treatment for CDI in unstable critically ill patients with extreme leukocytosis and diarrhea, along with treatment of other possible sources of sepsis as appropriate. Empiric treatment for CDI can usually be discontinued promptly, along with narrowing of other broad-spectrum antimicrobial coverage, if a sensitive *C. difficile* test is negative.

## 1. Background

*Clostridioides difficile* (formerly *Clostridium difficile*) infection (CDI) is a leading cause of healthcare-associated infections worldwide [1] and is associated with substantial excess mortality [2]. In the United States, *C. difficile* was responsible for almost half a million new infections and was associated with approximately 29,000 deaths in 2011 [3]. Critically ill patients are often susceptible to developing CDI due to immunosuppression, malnutrition, and use of antibiotics [4]. Presenting signs of CDI have not been well studied in large cohorts.

CDI is believed to be associated with a “particularly high leukocyte count (greater than 35,000/µL)” [5], and in a study of physicians prescribing patterns for patients being tested for CDI, leukocytosis was found to have the strongest influence on the decision to treat CDI empirically [6]. In this large observational study, our primary aims were to determine the prevalence of CDI in ICU patients with extreme leukocytosis and diarrhea and to assess the mortality rate for patients who test positive for CDI. Current Infectious Diseases Society of America guidelines recommend empiric treatment for CDI in those presenting with fulminant disease, characterized by hypotension or shock, ileus, or megacolon [7]. However, in patients presenting with sepsis, it can be challenging to determine the likelihood of CDI in comparison to other infectious etiologies. The findings of our study are relevant to providers deciding whether to initiate empiric treatment for CDI (alongside broad-spectrum antibiotics for other suspected pathogens) for unstable ICU patients with extreme leukocytosis and diarrhea, especially because delays in treatment of CDI may increase mortality [8].

## 2. Materials and Methods

This retrospective cohort study utilized the Medical Information Mart for Intensive Care III (MIMIC-III) database [9], which includes information from ICU patients admitted between 2001 and 2012 at the Beth Israel Deaconess Medical Center (BIDMC). The requirement for patient consent was waived by the Institutional Review Boards of BIDMC and the Massachusetts Institute of Technology.

In our primary analysis, we examined the prevalence of CDI in patients who were initially tested for CDI during their ICU stay. *C. difficile* toxin A and B enzyme immunoassay was used to detect CDI. Patients with myeloproliferative disorders such as leukemia or lymphoma were excluded because of difficulty interpreting leukocyte count in these populations. Since definitions of extreme leukocytosis have varied among previous studies from ≥25,000/µL to ≥50,000/µL [10,11,12], we chose a cut-off of ≥30,000/µL based on one of the most frequently cited studies [12]. Thus, patients who had a leukocyte count ≥30,000/µL on the day of CDI testing or within 3 days prior were considered to have extreme leukocytosis.

In our secondary analyses, we evaluated whether elevations in leukocyte count beyond 30,000/µL (e.g., ≥40,000/µL) were associated with increased risk of CDI. We stratified patients with extreme leukocytosis according to quintiles based on peak leukocyte count. The association between elevations in peak leukocyte count beyond 30,000/µL and higher rates of CDI were evaluated using one-way ANOVA. To ensure that extreme leukocytosis was not simply a late finding in the course of the disease, we assessed whether extreme leukocytosis within 3 days after CDI testing was more strongly associated with CDI. Additionally, we tested the prevalence of CDI in patients undergoing repeat testing for CDI who had not previously tested positive. Finally, multivariable logistic regression including age, sex, race, admission type (emergent vs. urgent, medical vs. surgical), and extreme leukocytosis was used to test for independent associations, and mortality rates were compared across study populations. A *p*-value of ≤0.05 was the criterion of significance. All statistical analyses were done using Stata (v14.2, StataCorp LLC, Lakeway, TX, USA).

## 3. Results

**Figure 1:**
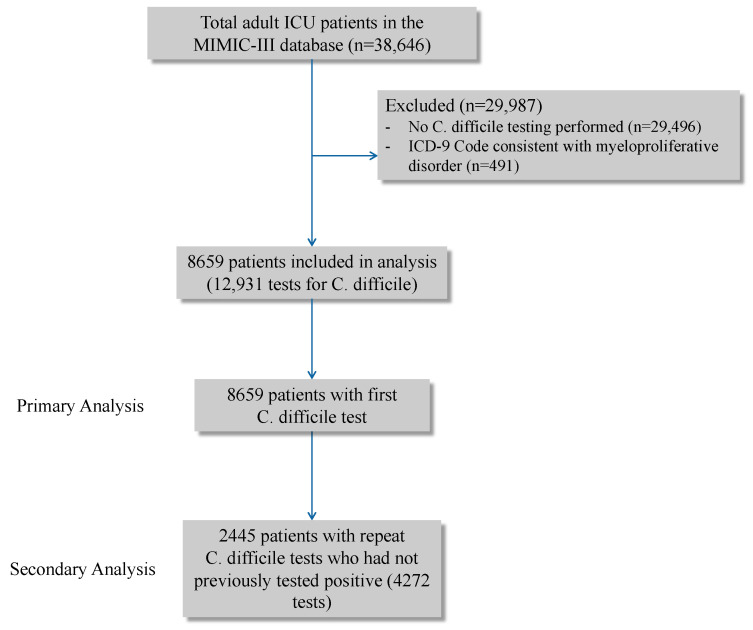
Study flow.

Of the 38,646 adults in the database, 8659 had diarrhea and underwent first-time CDI testing and met the inclusion criteria (Figure 1). Among these patients, 685 tested positive, corresponding to a prevalence of 7.9% (95% CI, 7.4–8.5%; Table 1). Assuming those not tested for CDI did not have CDI, and taking into consideration the 253 patients who tested positive on subsequent CDI testing after a negative result on the first test, the overall prevalence of CDI in the ICU at the medical center was 2.3% (898/38,646).

Of the 534 patients with extreme leukocytosis within three days prior to CDI testing as well as diarrhea, 80 had CDI, corresponding to a prevalence of 15.0% (95% CI, 12.1–18.3%). Elevations in peak WBC count beyond 30,000/µL (e.g., ≥40,000/µL) were not associated with increased prevalence of CDI (*p* = 0.84) compared to WBC ≥ 30,000. Prevalence of CDI was similar between those who developed extreme leukocytosis within three days after *C. difficile* testing and those who had extreme leukocytosis before *C. difficile* testing (15.9% vs. 15.0%, *p* = 0.66). The prevalence of CDI in patients with extreme leukocytosis and diarrhea undergoing repeat testing during the same or subsequent ICU stay who had not previously tested positive was 6.1% (95% CI, 3.2–9.2%). On multivariable logistic regression, extreme leukocytosis was an independent predictor of CDI (aOR 2.1, 95% CI 1.7–2.8, *p* < 0.001). Medical admission as opposed to surgical (aOR 1.22, 95% CI 1.03–1.43, *p* = 0.02) and age (aOR 1.019, 95% CI 1.013–1.024 for every year of age, *p* < 0.001) were also significant predictors of CDI. Race, sex, and emergent admission (as opposed to urgent) were not significant predictors of CDI on multivariable regression.

The mortality rate among patients with diarrhea who were tested for *C. difficile* was 15.2% (95% CI, 14.4–16.0%). Those with diarrhea who were tested for *C. difficile* and who also had extreme leukocytosis had a mortality rate of 27.0% (95% CI, 23.2–30.7%). The subgroup of patients with the highest mortality rate was the subgroup of patients that had extreme leukocytosis, had diarrhea, and tested positive for CDI (33.8%; 95% CI, 23.2–44.3%).

## 4. Discussion

In this large retrospective cohort study, we found that the prevalence of CDI in those with extreme leukocytosis and diarrhea was 15.0%, and the mortality rate for those with extreme leukocytosis and diarrhea who tested positive for CDI exceeded 30%.

The overall prevalence of CDI in our study of 2.3% was consistent with the 2% prevalence of CDI in the ICU previously reported in a recent large systematic review [4]. Our findings contrast somewhat with a smaller study evaluating 53 general medical patients with leukocyte count greater than 30,000/µL, which found that approximately 25% (13 patients) had CDI [12]. Both our study and their study used *C. difficile* toxin enzyme immunoassay to detect CDI; however, the *C. DIFFICILE* tests in their study only detected toxin A, and the patients in our study were critically ill.

One limitation is that our data were derived from a single center and were collected over a number of years. Our sample thus represents the average CDI rate across the study period. Additionally, while inpatients often have bloodwork performed every day (as was the case for the majority of patients in our study), some patients had fewer days of bloodwork available if they were admitted from home just prior to their *C. difficile* test. Outpatient bloodwork (for example, performed at a clinic prior to admission) was not available in the database. Furthermore, we did not include patients with CDI who did not have diarrhea in our cohort. Finally, our institution used *C. difficile* toxin A and B enzyme immunoassays, which may underestimate the prevalence of CDI compared to nucleic acid amplification testing [13,14]. Had our study been performed using nucleic acid amplification testing, which has greater sensitivity but also a higher probability of detecting *C. difficile* carriers without infection, our estimated prevalence of CDI would likely have been higher.

## 5. Conclusions

CDI is relatively common among critically ill patients with extreme leukocytosis and diarrhea and is associated with very high mortality. The results of our study provide preliminary support for the consideration of empiric treatment for CDI in unstable critically ill patients with extreme leukocytosis and diarrhea while awaiting stool testing results, particularly at institutions where rapid *C. difficile* testing is not available, along with therapy for other possible sources of sepsis. Empiric treatment for CDI can usually be discontinued promptly, along with narrowing of other broad-spectrum antimicrobial coverage, if a sensitive *C. difficile* test is negative. Additional studies are needed to determine whether, as for other forms of sepsis, empiric treatment of patients suspected to have sepsis due to CDI may improve outcomes.

## Figures and Tables

**Table 1:** Baseline characteristics of patients in the intensive care unit with diarrhea with and without *Clostridioides difficile* infection on initial testing.

Variables	Negative for *C. difficile* (n = 7974)	Positive for *C. difficile* (n = 685)	*p*-Value *
Age, mean ± SD	65.1 ± 16.7	70.0 ± 15.9	<0.001
Male, n (%)	4266 (53.5%)	366 (53.4%)	0.97
Surgical ^a^, n (%)	3588 (45.3%)	268 (39.4%)	0.003
Ethnicity, n (%)			0.051
White	5709 (71.6%)	527 (76.9%)	
Black	736 (9.2%)	50 (7.3%)	
Hispanic	238 (3.0%)	15 (2.2%)	
Other	411 (5.2%)	27 (3.9%)	
Unknown	880 (11.0%)	65 (9.6%)	
Admission Type, n (%)			0.28
Planned	664 (8.3%)	49 (7.2%)	
Urgent/Emergent	7310 (91.7%)	636 (92.8%)	
Peak leukocyte count prior to *C. difficile* testing ^b^, mean ± SD	15.5 ± 8.6	17.8 ± 11.0	<0.001

^a^ Admission to a surgical intensive care unit during the hospital stay. ^b^ Within 3 days prior to *Clostridioides difficile* testing, including the day of the test result. * *p*-value ≤ 0.05 was considered statistically significant.

## Data Availability

The data presented in this study are openly available in the MIMIC-III repository at https://doi.org/10.13026/C2XW26.
